# Soil nutrient concentrations influence micronutrient concentrations in *Eragrostis curvula* seeds

**DOI:** 10.1371/journal.pone.0322214

**Published:** 2025-04-29

**Authors:** Nqobile Motsomane, Rob Slotow, Anathi Magadlela

**Affiliations:** 1 School of Life Sciences, College of Agriculture, Engineering, and Science, University of KwaZulu Natal (Westville Campus), Private Bag X54001, Durban, South Africa; 2 Oppenheimer Fellow in Functional Biodiversity, Centre for Functional Biodiversity, School of Life Sciences, College of Agriculture, Engineering, and Science, University of KwaZulu Natal (Pietermaritzburg Campus), Private Bag X01, Scottsville, South Africa; 3 Department of Biological and Agricultural Sciences, Faculty of Natural and Applied Sciences, Sol Plaatje University, Private Bag X5008, Kimberley, South Africa; National Museums of Kenya, KENYA

## Abstract

Food insecurity often results in malnutrition, manifesting as micronutrient deficiencies that disproportionately affect children under five, impairing their cognitive and physical development. While staple crops supply necessary calories and basic nutrients required for life sustenance, they frequently lack essential micronutrients needed for overall health, necessitating alternative food sources to address food insecurity and malnutrition. This study investigated the potential of *Eragrostis curvula* seeds as a micronutrient-rich addition to existing food systems and used analyses of covariance to evaluate the influence of soil characteristics on seed nutrient concentrations between two grassland sites in South Africa’s Gauteng province: Jameson Park and Kaydale. Populations of *E. curvula* were identified in Jameson Park and Kaydale, Heidelberg, where rhizosphere soils from selected plants and seeds were harvested for nutrient concentration and soil characteristics analysis. *Eragrostis curvula* seeds were rich in essential micronutrients, including phosphorus (5041.5–5921.7 mg/kg), iron (72.2–145.4 mg/kg), potassium (4490.5–5531.2 mg/kg), zinc (44.9–65.4 mg/kg), copper (8.6–10.2 mg/kg), calcium (2978.4–16339.1 mg/kg), magnesium (2265.7–2538.4 mg/kg), and manganese (130.2–141.8 mg/kg). Significant site-specific variations were observed: seeds from Jameson Park had higher potassium and calcium concentrations, while Kaydale seeds had higher zinc and iron concentrations. Soil analyses revealed no significant differences in phosphorus, nitrogen, zinc, copper, exchange acidity, or total cation concentrations between the sites. However, calcium levels were significantly higher in Jameson Park soils, while Kaydale soils showed higher potassium, magnesium, and manganese concentrations. Soil nutrient concentrations were found to have a significant influence on seed nutrients. These findings emphasise the potential of *E. curvula* seeds to improve food security and alleviate micronutrient deficiencies, particularly among vulnerable populations such as young children.

## 1 Introduction

Food insecurity is a global issue exacerbated by social inequalities, inflation, unemployment, and the Ukraine war, with Africa experiencing a rapid increase in hunger [1]. In South Africa, food insecurity is further exacerbated by high unemployment, poverty, ongoing energy crisis, and rising living costs [2]. These challenges have led to widespread food access issues, with 2.6 million households reporting inadequate food access and an additional 1.1 million facing severe food shortages [2]. Efforts to improve food security have led to increased reliance on affordable and high-energy-density staple cereal grains [1;3], which can be seen by the dominance of wheat, rice, and maize in human diets globally from 1961 to 2009 [4]. Though dietary staples such as maize contribute significantly to daily caloric intake, they lack essential micronutrients such as iron (Fe) and magnesium (Mg) [5]. The continuous consumption of food deficient in essential micronutrients can lead to micronutrient deficiencies, particularly affecting vulnerable groups such as children and pregnant women, who are more susceptible to the consequences of inadequate nutrition [6].

According to the World Health Organisation (WHO), 45% of deaths in children under the age of 5 are due to undernutrition, 149 million children under 5 are estimated to be stunted and 45 million children are estimated to be wasted [6]. Approximately 98 million preschool-aged children in Sub-Saharan Africa are affected by micronutrient deficiencies, which have been shown to negatively impact cognitive development and physical growth [7]. Micronutrient deficiencies, such as iron deficiency, lead to anaemia, affecting red blood cell production and causing health complications [8]. Zinc deficiencies impair cognitive and motor functioning, which directly impact child development [9]. The co-occurrence of micronutrient deficiencies with communicable and non-communicable diseases further exacerbates health outcomes [10], particularly in countries like South Africa, where high rates of HIV and tuberculosis (TB) prevail [11]. The co-occurrence of HIV infection and malnutrition weakens the immune system, aggravating health issues in children [12]. While South Africa has improved its neonatal survival rates, the under-5 mortality rate remains stagnant [11], highlighting the need for targeted interventions. Addressing micronutrient deficiencies could be a crucial step in improving child survival and overall health outcomes.

Promoting the cultivation and consumption of non-staple grains, such as *Eragrostis* species that produce nutrient-dense seeds, can help address micronutrient deficiencies [13]. *Eragrostis tef* has been reported to mitigate iron deficiency [14] and play a role in the prevention of non-communicable diseases [15]. While the nutritional benefits of *E. tef* have been well-documented in literature [16–17], *Eragrostis curvula* remains underexplored in the context of micronutrient supplementation and food insecurity alleviation. *Eragrostis curvula*, a perennial relative of the annual *E. tef* [18], may offer similar nutritional benefits, owing to its congeneric relationship with *E. tef*. Moreover, *E. curvula* provides the advantage of being a perennial grass that produces seeds twice a year, thereby enhancing its potential yield and increasing its value as a reliable perennial grain crop [19]. *Eragrostis curvula* has also been consumed during famine periods [20], suggesting its potential to contribute as a food source. Though recent studies, such as [19], provide insights into the nutrient composition of *E. curvula* seeds compared to *E. tef*, research addressing the use of *E. curvula* seeds to alleviate micronutrient deficiencies in impoverished communities remains scarce.

Furthermore, the influence of soil characteristics on the micronutrient profile of *E. curvula* seeds is an area that has yet to be fully explored. This gap highlights the need for studies focusing on both the nutritional potential of *E. curvula* and the environmental factors that affect its nutrient composition to address food insecurity and micronutrient deficiencies alleviation. Heidelberg, located within the Lesedi Local Municipality, is significantly impacted by socio-economic challenges, including an unemployment rate of 43.9%, 12% of households having no income, and 28.2% of the population living below the food poverty line [21]. This area is home to 10,687 children under the age of five, many of whom are at heightened risk of micronutrient deficiencies due to poverty and limited access to diverse diets [21]. Promoting *E. curvula* as a food source in this context offers substantial benefits by providing a nutrient-rich alternative to traditional staples, potentially mitigating the nutritional deficits experienced in impoverished households. Beyond individual health benefits, integrating *E. curvula* into local food systems can contribute to broader goals of enhancing food security, diversifying agricultural options, and supporting climate-resilient practices. These actions not only align with the United Nations’ Sustainable Development Goals but also support Heidelberg’s aspirations for sustainable economic and agricultural development [21,22]. This study investigated the potential of *E. curvula* seeds as a micronutrient-rich addition to existing food systems and evaluated the influence of soil characteristics on seed nutrient concentrations. The objectives of this study include determining the soil characteristics in *E. curvula* planted fields, harvesting *E. curvula* seeds, and conducting a nutrient analysis of the seeds. We hypothesised that *E. curvula* seeds will contain high concentrations of essential micronutrients, highlighting their potential for inclusion in food systems aimed at addressing micronutrient deficiencies. Furthermore, variations in seed nutrient concentrations would be shaped by differences in soil characteristics at Jameson Park and Kaydale locations.

## 2 Materials and methods

### 2.1 Collection sites

**Fig 1:**
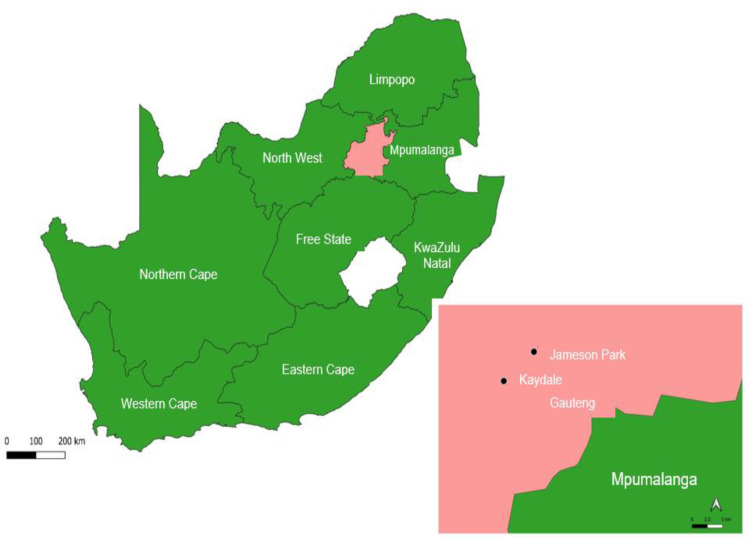
Map of the collection sites. The provincial shapefile was drawn in the South African National Geo-Spatial Information (NGI) website and imported into QGIS, where the coordinate reference system was verified. The map was styled, labelled, and supplemented with a basemap before being finalized in the Layout Manager and exported as an image. The map was drawn on 06 February 2025.

*Eragrostis curvula* populations were identified in Jameson Park (26°26’31.7 “S; 28°26’01.4”E) and Kaydale (26°29’12.4”S; 28°23’02.1”E), Heidelberg, South Africa (Fig. 1). Heidelberg is in Gauteng, which consists of savanna and grassland ecosystems [23]. Heidelberg is in Gauteng, which consists of savanna and grassland ecosystems [23]. In Gauteng, Heidelberg experiences summer rainfall followed by dry winters [24]. Annual temperatures range from 3–25°C, and precipitation ranges from 600–700 mm per annum [24,25]. Jameson Park and Kaydale, both situated in Heidelberg, Gauteng Province, exhibit distinct edaphic characteristics. Jameson Park soils had a clay content of 29% and an organic carbon content of 1.2%, whereas Kaydale soils contained 35% clay and 1.8% organic carbon. The soils in Jameson Park were red and sandy, with cattle grazing in the vicinity, which may have contributed to nutrient inputs through manure deposition. In contrast, Kaydale soils were a sandy brown colour. These differences in soil texture, organic carbon content, and land use likely influenced nutrient availability and uptake, thereby affecting seed nutrient concentrations.

### 2.2 Soil and seed collection

Forty randomly selected mature *E. curvula* plants were sampled from each site and removed with the soil using a shovel. The plants were separated from the soil, placed on sheets of paper on metal trays, and sundried for five days. After five days, seeds were removed from the inflorescence and cleaned manually to remove foreign materials and immature seeds. The seeds collected from all plants per site were mixed and weighed, resulting in two batches weighing 64 g for Jameson Park and 88 g for Kaydale. The seeds were oven-dried at 65°C until a constant weight was maintained and ground into a fine powder. Six 0.25 g samples per site were sent for ICP AES: Basic chemistry (potassium, magnesium, copper, iron, manganese, calcium, zinc, and phosphorus) analysis at the Central Analytical Facilities, Stellenbosch University, South Africa. The remaining soils from all sampled plants per site were mixed to form two composite soil samples placed in sterile Ziploc bags and stored at 4°C until soil nutrient analysis.

### 2.3 Soil nutrient analysis

Six soil subsamples of 300g from each site were sent to the Analytical Services Unit, KwaZulu Natal Department of Agriculture and Rural Development, Cedara, South Africa, for soil characteristic analysis (nutrient concentrations, total cation concentrations, exchange acidity, and pH). The characteristic soil analysis was performed per protocols [26] explained. Ambic-2 solution containing 0.25M NH_4_CO_3_, 0.01M Na_2_EDTA, 0.01M NH_4_F, and 0.05 g/L superfloc (N100) was adjusted to pH 8 using concentrated ammonia solution and used to extract phosphorus, potassium, zinc, and copper. The extracts were filtered using Whatman no.1, and a 2 ml filtrate aliquot was used to determine the phosphorus concentration using a modified protocol of [27] molybdenum blue procedure. The potassium concentration was determined by diluting 5 ml aliquot of the filtrate with 20 ml de-ionised water using atomic absorption, and the remaining undiluted filtrate was used to determine the zinc, copper, and manganese concentration using atomic absorption. The magnesium and calcium concentrations were determined by stirring sample cups containing 25 ml of soil sample and 25 ml of 1M KCl solution in a multiple stirrer (400 rpm) for 10 minutes. The stirred mixture was filtered with Whatman no.1 paper. Five mililitres of the filtrate was diluted with 20 ml 0.0356M SrCl_2_, and calcium concentrations were determined using atomic absorption. Soil nitrogen concentration was measured using the Automated Dumas dry combustion method with a LECO CNS 2000 (Leco Corporation, USA). Soil samples were weighed in a ceramic crucible, and 0.5 g vanadium pentaoxide was used as a combustion catalyst. The crucible was placed in a horizontal furnace and burned in a stream of oxygen at 1350 °C, and soil nitrogen was measured as N_2_ in a thermal conductivity cell. Soil pH was determined by mixing 10 ml of soil sample and 25 ml of 1M KCl in sample cups and stirring in a multiple stirrer at 400 rpm for 5 minutes. The suspension was left to rest for 30 minutes, and the pH was measured using a gel-filled combination glass electrode while stirring.

### 2.4 Statistical analysis

The statistical software R (version 3.6.2) was used to test for differences in the nutrient concentrations of *E. curvula* seeds collected from Jameson Park and Kaydale and the soil characteristics of Jameson Park and Kaydale using an Independent Samples T-test. Assumptions of normality and homogeneity of variances were tested using the Shapiro-Wilk normality test and Levene’s test, respectively. The leveneTest() from the car package was used to conduct Levene’s test [28] and shapiro.test() was used for the Shapiro-Wilk normality test [29]. When the data distribution was not normal, a log transformation was applied to the data to improve normality, and the Welch’s T-test was performed to account for unequal variances [29]. The soil nutrient concentrations were converted from mg/L to mg/kg and a One-Way analysis of covariance (ANCOVA) was used to determine the influence of site (categorical independent variable) and soil concentration (continuous covariate) on seed nutrient concentration (dependent variable) using the lm function. The scatterplot showing the relationship between soil and seed nutrient concentrations was produced using ggplot2.

## 3 Results

### 3.1 Soil nutrient concentrations

**Fig 2:**
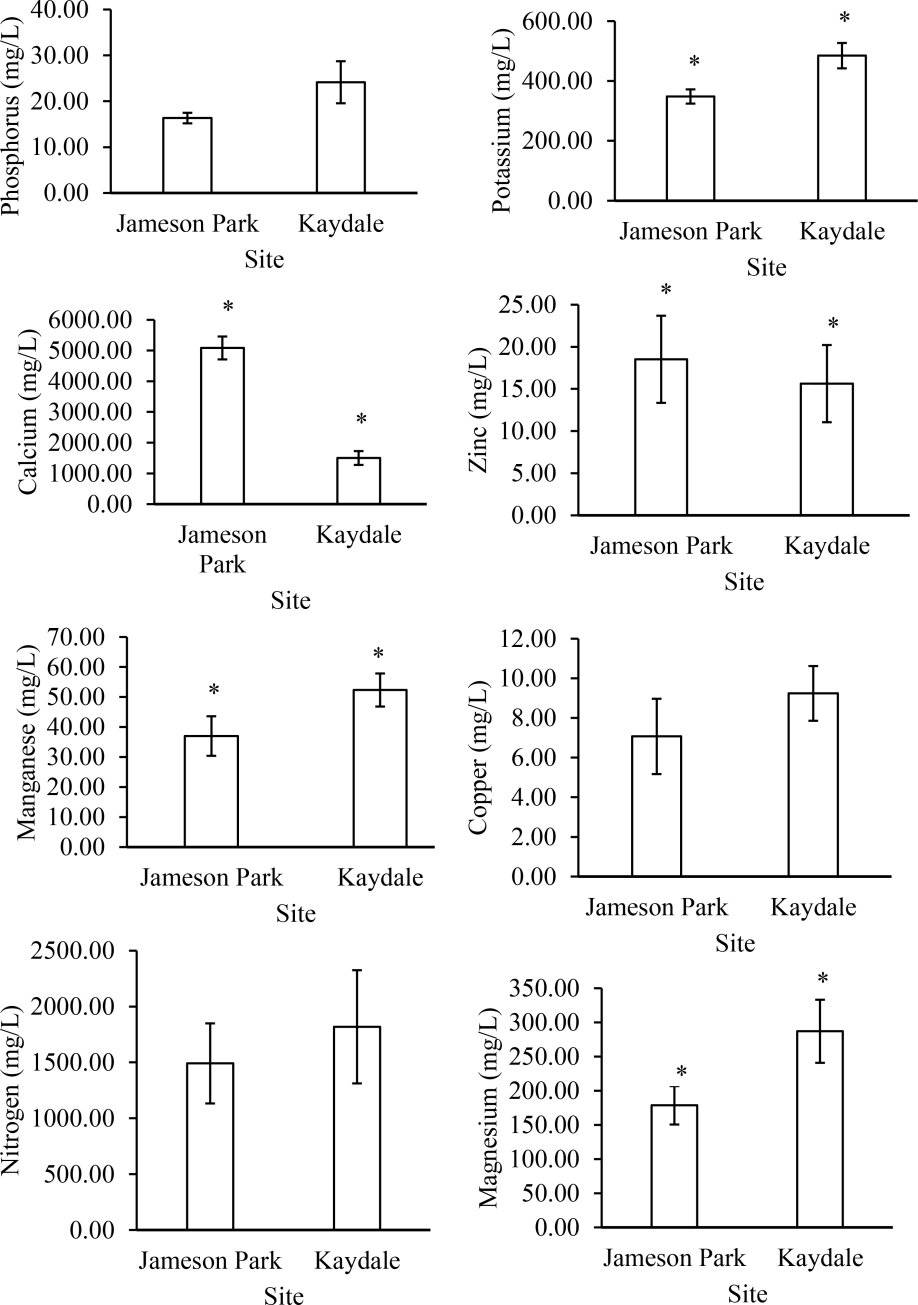
Soil characteristics of Jameson Park and Kaydale soils in Heidelberg, Gauteng. Bars represent means and error bars represent standard deviation (n = 12). Asterisks (*) denote significant differences between the means, as determined by an Independent Samples T-test (p ≤ 0.05).

**Fig 3:**
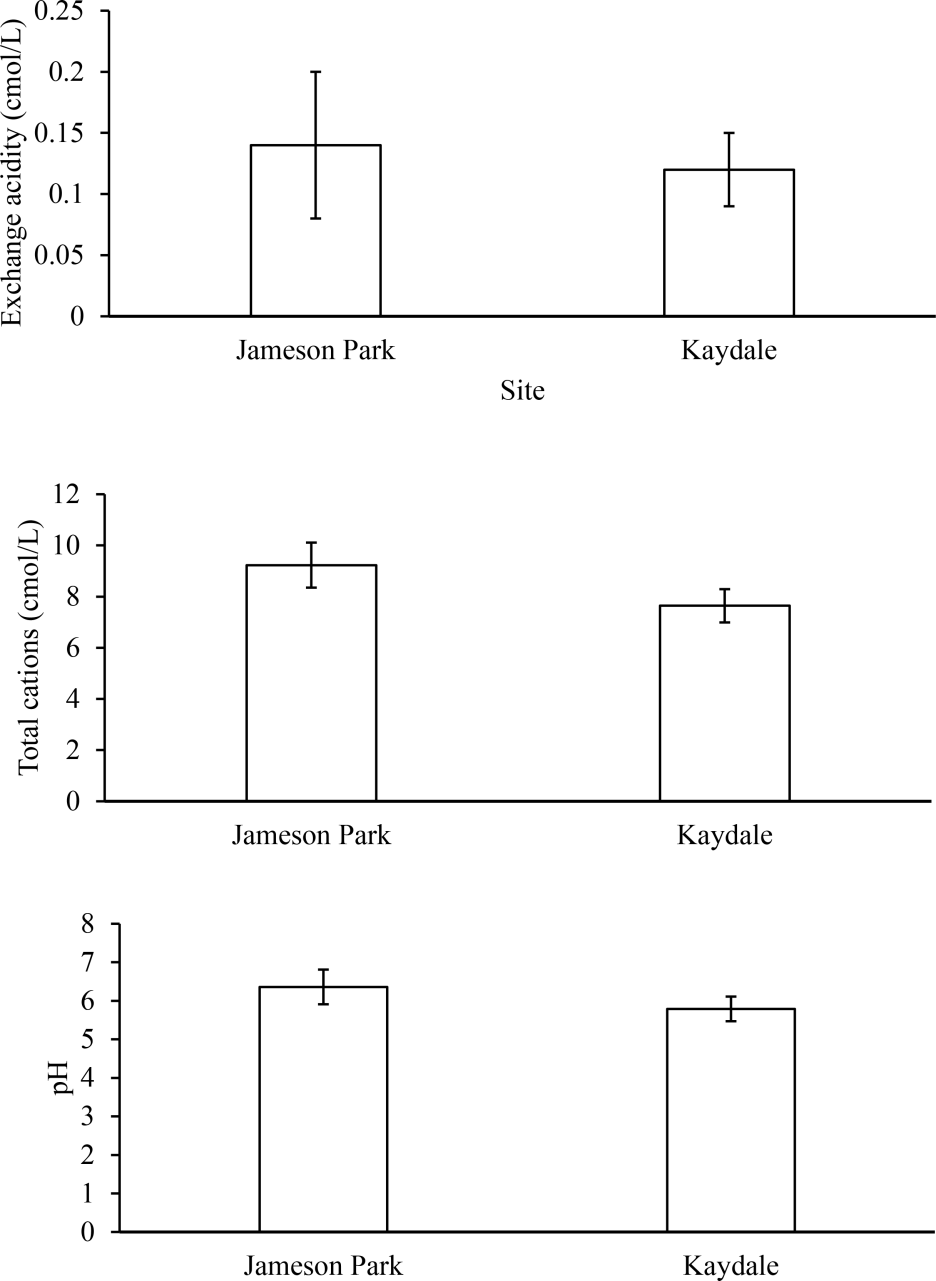
Relative acidity of Jameson Park and Kaydale soils in Heidelberg, Gauteng. Bars represent means and error bars represent standard deviation (n = 12). Asterisks (*) denote significant differences between the means, as determined by an Independent Samples T-test (p ≤ 0.05).

The soil characteristics of Jameson Park and Kaydale soils are represented in Fig 2. Although the phosphorus, nitrogen, and copper concentrations were higher in Kaydale soils than in Jameson Park soils, these differences were not statistically significant. In contrast, the potassium, magnesium, and manganese concentrations were significantly higher in Kaydale soils, whereas the calcium concentration was significantly higher in Jameson Park soils. The relative acidity of Jameson Park and Kaydale soils is represented in Fig 3. The exchange acidity, total cations, pH, and zinc concentrations were higher in Jameson Park soils, but the differences were also insignificant. Figs 2 and 3 highlight variations in the soil characteristics of Jameson Park and Kaydale, which may influence seed nutrition differently.

### 3.2 Seed nutrient composition

**Fig 4:**
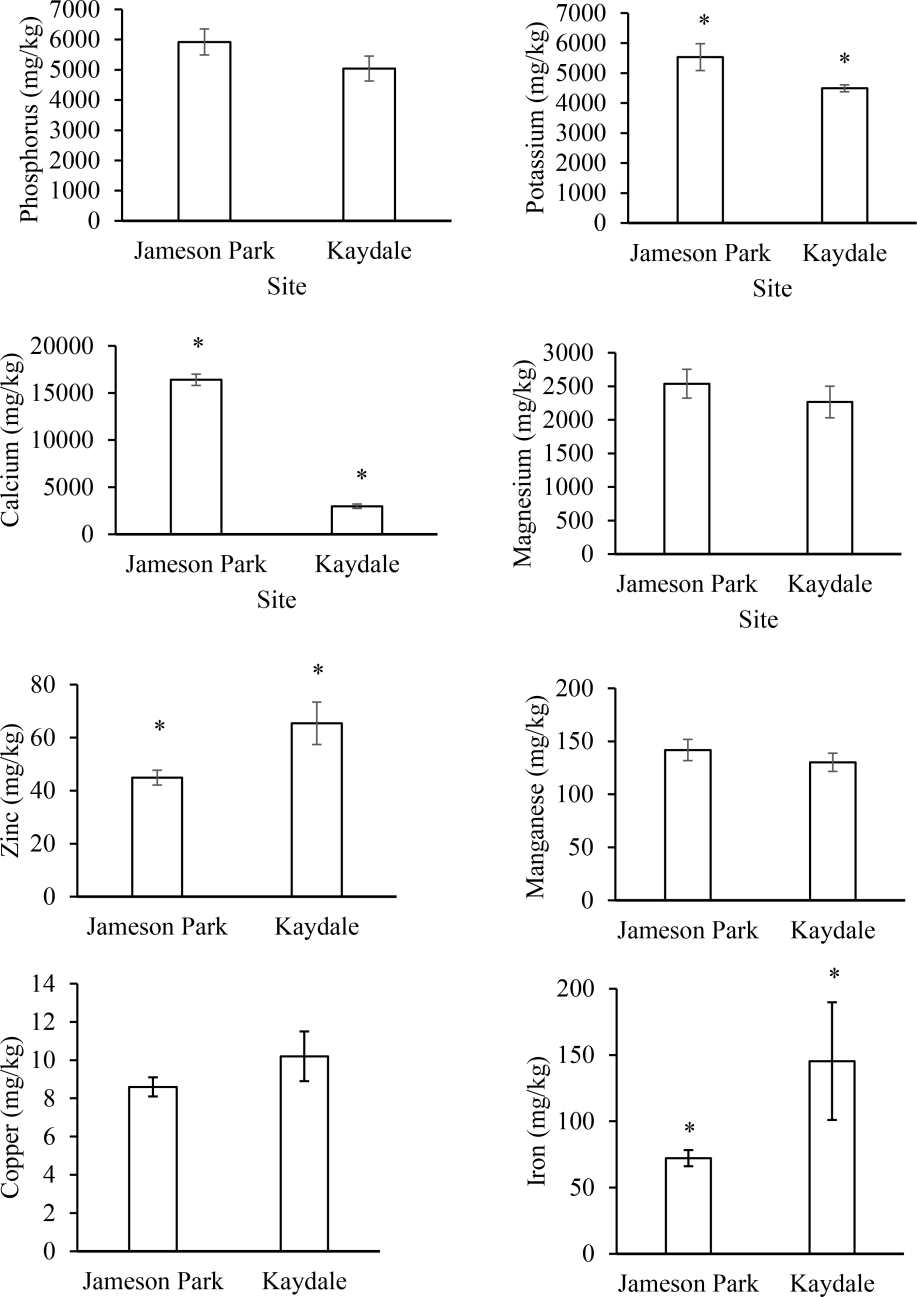
Nutrient concentrations of *Eragrostis curvula* seeds harvested from Jameson Park and Kaydale, Heidelberg, Gauteng. Bars represent means and error bars represent standard deviation (n = 12). Asterisks (*) indicate statistically significant differences, as determined by an Independent Samples T-test (p ≤ 0.05).

The nutrient concentrations of seeds harvested from *E. curvula* plants growing in Jameson Park and Kaydale are represented in Fig 4. *Eragrostis curvula* seeds from both localities exhibited high concentrations of essential micronutrients. However, seeds from Jameson Park had higher concentrations of most micronutrients compared to those from Kaydale, with potassium and calcium concentrations being significantly higher. Although not statistically significant, phosphorus, magnesium, and manganese concentrations were also higher in Jameson Park seeds. Conversely, the iron concentration was significantly higher in Kaydale seeds, and while not significant, copper concentrations were also higher in Kaydale seeds compared to Jameson Park.

### 3.3 Effects of soil nutrients on seed nutrients

**Fig 5:**
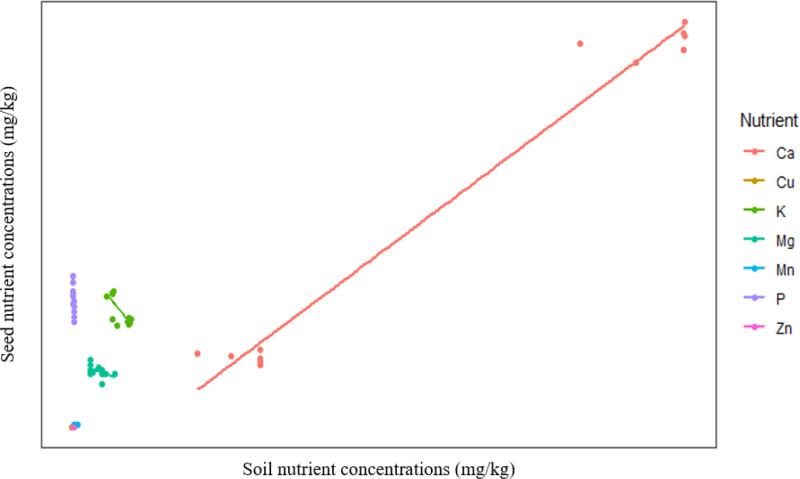
Scatter plot showing the relationship between soil and seed nutrient concentrations. Points represent individual observations, with colours indicating different nutrient groups from a One-way ANCOVA. The fitted regression line illustrates the trend of seed nutrient variation in response to soil nutrient availability.

**Fig 6:**
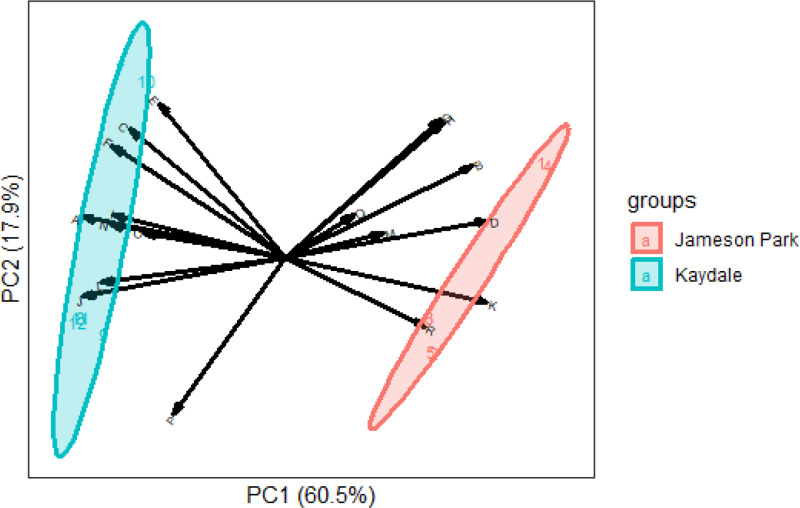
Correlations between *Eragrostis curvula* seed nutrients and soil nutrient concentrations from Jameson Park and Kaydale, Heidelberg, Gauteng. The letters represent the following: A: Seed calcium, B: seed phosphorus, C: seed iron, D: seed potassium, E: seed copper, F: seed zinc, G: seed magnesium, H: seed manganese, I: soil phosphorus, J: soil potassium, K: soil calcium, L: soil magnesium, M: soil zinc, N: soil manganese, O: soil copper, P: soil nitrogen, Q: soil exchange acidity, R: soil total cation concentration and S: soil pH. Principal component analysis.

The relationship between soil and seed nutrient concentrations is represented in Fig 5. Soil nutrient concentrations were a significant factor influencing *E. curvula* seed nutrients (F= 1485.8, p<0.05). Relations between soil and seed nutrient concentrations of *E. curvula* seeds harvested from Jameson Park and Kaydale, Heidelberg, Gauteng, are represented in Fig 6. The first PCA explained 60.5% of the total variation, while PCA2 explained 17.9%. The separation of clusters indicates that the nutrient concentrations of *E. curvula* seeds differed between the two collection sites, Jameson Park and Kaydale. Additionally, the PCA showed relationships between soil properties and seed nutrients. Groups closer together showed a high correlation.

## 4 Discussion

*Eragrostis curvula* seeds harvested from Jameson Park and Kaydale, Heidelberg, contain high concentrations of essential micronutrients, demonstrating their potential as a valuable addition to existing food systems. These seeds could help address micronutrient deficiencies, particularly in vulnerable populations, such as children under the age of five*.* The daily recommended concentrations for iron and zinc are 57 mg/kg and 41 mg/kg, respectively [30]. In this study*, E. curvula* seeds from both locations exceeded these recommended concentrations (iron 72.2–145.4 and zinc 44.9–65.4 mg/kg), suggesting their potential to supplement daily dietary requirements for these essential micronutrients*.* The inability to meet the required levels of iron has been reported to increase the risk of infections and the prevalence of iron deficiency anaemia [31], which manifests as fatigue, inflammatory bowel disease, and impaired cognitive development [32,33]*.* Similarly, insufficient zinc nutrition has been associated with reduced resistance to diseases [34], often resulting in delayed wound healing, growth retardation, and a weakened immune response [35]. Consuming *E. curvula* seeds could help children meet their recommended iron and zinc intake, thereby reducing the prevalence of iron deficiency anaemia, enhancing neurophysiological outcomes, and decreasing the incidence of infections, such as acute lower respiratory tract infections [31,34].

In South Africa, staple foods, such as maize, sugar, tea, milk, and brown bread, dominate daily diets [36]. Wheat, the primary component of brown bread, contains 26 mg/kg of zinc, 32.76 mg/kg of iron, 34.7 mg/kg of copper, and 5.94 mg/kg of manganese [37]. The present study revealed that the zinc, iron, and manganese concentrations in *E. curvula* seeds were higher than those reported for wheat. Given that South African children’s intake of energy, calcium, iron, and zinc remains below two-thirds of the Recommended Dietary Allowances [36], incorporating *E. curvula* seeds into widely consumed staple-based food systems like maize and wheat could help address nutritional gaps. Furthermore, supplementation with calcium, phosphorus, and magnesium has been reported to improve bone mass and help prevent the development of osteoporosis later in life [38]. Though the consumption of *E. curvula* is promising, challenges such as consumer acceptance should be considered to ensure successful integration into existing food systems. While studies on the nutrient composition of *E. curvula seeds* for human consumption remain limited, one prior study [19] reported on the macro- and micronutrient composition of these seeds, noting moderate levels of calcium, phosphorus, and potassium, as well as lower concentrations of iron and zinc compared to those observed in the current study.

The differences in micronutrient concentrations between seeds harvested from Jameson Park and Kaydale suggest that variations in soil characteristics*,* as demonstrated by the significant differences in the potassium, magnesium, manganese and calcium concentrations. Similar findings have been reported for *E. tef*, where seed nutrient variability was attributed to differences in soil and climatic conditions of the harvesting sites [39]. Furthermore, soil macronutrients were shown to influence macronutrient concentrations in *Peucedanum oreoselium* seeds [40], and edaphic and climatic factors significantly affected nutrient composition in *Asterothamnus centraliasiaticus* seeds [41]. These studies support the conclusion that soil properties and environmental factors likely contributed to the differences in calcium, iron, potassium, and zinc concentrations observed in *E. curvula* seeds from Jameson Park and Kaydale. The principal component analysis (PCA) confirmed that *E. curvula* seeds from the two locations formed distinct clusters and soil nutrient concentrations were found to have a significant influence on seed nutrients, highlighting the influence of unique soil characteristics and environmental conditions on seed nutrient composition*.*

While this study focused on micronutrient concentrations, it did not assess other essential nutrients, such as vitamins A and D, which are crucial for growth and development. Future research should expand on this work by increasing sample sizes, analysing additional micro- and macronutrients, and including more localities to strengthen the understanding of *E. curvula’s* potential in enhancing food and nutrition security*.* These efforts could address current limitations, such as the limited scope of nutrient analysis and the lack of data on environmental variability, thereby providing a more comprehensive picture of the factors influencing *E. curvula* seed nutrition. Overall, this study underscores the need for further exploration of the combined effects of soil properties and climatic conditions on *E. curvula* seed nutrition.

## Conclusions

This study highlights that *E. curvula* seeds are nutrient-dense and well-suited for incorporation into existing food systems. Notably, a dish prepared using *E. curvula* seeds demonstrated comparable taste, texture, appearance, and acceptability to the traditional Injera made with *E. tef* [19], indicating similar sensory attributes. The findings suggest that *E. curvula* holds significant potential to address food insecurity and micronutrient deficiencies, enhance nutritional diversity, and promote sustainability in food systems, offering a promising alternative to traditional crops for improving global food security.

## Supporting information

S1 Table Nutrient concentrations (mg/kg) of *Eragrostis curvula* and *Eragrostis tef* seeds from the current study compared to values reported in the literature. Values from Ghebrehiwot et al. (2016) were converted from mg/100g to mg/kg.
(DOCX)
