# Supplementary material for: Soil nutrient concentrations influence micronutrient concentrations in Eragrostis curvula seeds
Source: PLoS One. 2025 Apr 29;20(4):e0322214. doi: 10.1371/journal.pone.0322214 (PMC12040202; doi:10.1371/journal.pone.0322214)
Supplement: S1 Table — Values from Ghebrehiwot et al. (2016) were converted from mg/100g to mg/kg. (DOCX) [file pone.0322214.s001.docx]

| **Nutrient concentrations (mg/kg)** | ***Eragrostis curvula*** | | | ***Eragrostis tef*** | | | |
| --- | --- | --- | --- | --- | --- | --- | --- |
|  | **Jameson Park** | **Kaydale** | **Ghebrehiwot et al. (2016)** | **Inglett et al. (2015)** | **Habte et al. (2020)** | **Nyachoti et al. (2021)** | **Ghebrehiwot et al. (2016)** |
| Phosphorus | 5921.7 | 5041.5 | 4.1 | 4290 | n/a | n/a | 4.2 |
| Potassium | 5531.2 | 4490.5 | 5.8 | 4270 | 2883-3071 | n/a | 4.2 |
| Calcium | 16339.1 | 2978.4 | 2.2 | 1800 | 1211-1739 | 1210 | 1.9 |
| Magnesium | 2538.4 | 2265.7 | 1150 | 1840 | 2478-2552 | 1400 | 3541.8 |
| Zinc | 44.9 | 65.4 | 514.3 | 36.3 | 35-85 | n/a | 373.0 |
| Manganese | 141.8 | 130.2 | 1149.4 | n/a | 56-99 | 71 | 3541.8 |
| Copper | 8.6 | 10.2 | 100.2 | n/a | 6.7-42 | 4.8 | 42.7 |
| Iron | 72.2 | 145.4 | 845.3 | 76.3 | 212-239 | 159 | 507.8 |
